# Tailoring mRNA lipid nanoparticles for antifungal vaccines

**DOI:** 10.1371/journal.ppat.1013091

**Published:** 2025-04-28

**Authors:** Yeqi Li, Richard B. Meagher, Xiaorong Lin

**Affiliations:** 1 Department of Microbiology, University of Georgia, Athens, GeorgiaUnited States of America; 2 Department of Genetics, University of Georgia, Athens, GeorgiaUnited States of America; Vallabhbhai Patel Chest Institute, INDIA

## Abstract

Vaccination is one of the most effective public health measures for preventing and managing infectious diseases. Despite intensive efforts from the relatively small medical mycology community, developing effective vaccines against invasive fungal infections remains a scientific challenge. This is predominantly due to large antigenic repertoires, complicated life cycles, and the capacity of fungal pathogens to evade the host immune system. Additionally, antifungal vaccines often need to work for at-risk individuals who are immunodeficient. We anticipate that the success of mRNA vaccines against severe acute respiratory syndrome coronavirus 2 (SARS-CoV-2) and its exploration for various infectious diseases and cancers will usher a new wave of antifungal vaccine research. Herein, we discuss recent advancements and key scientific areas that need to be explored to actualize the development of effective antifungal mRNA vaccines.

## 1. What are the major advantages and barriers of developing mRNA vaccines against fungal infections?

Several recent reviews have discussed the progress of fungal vaccine development using conventional vaccine platforms, such as whole-cell vaccines (live, attenuated, or inactivated cells) and subunit protein vaccines [1–4]. None of these experimental vaccines has been approved for clinical use for humans yet, which is likely due to various obstacles such as safety, duration, and efficacy in immunocompromised individuals [5–9]. Thus, it is important to explore additional platforms such as mRNAs for fungal vaccine development.

**Fig 1:**
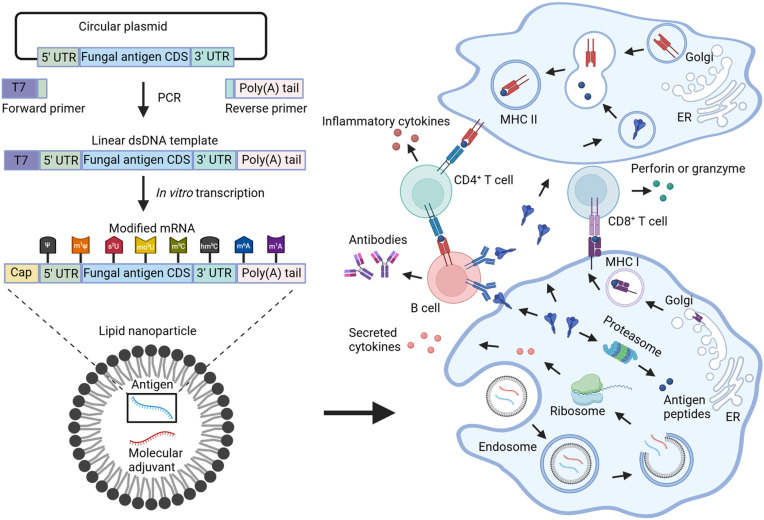
mRNA-LNP vaccine model. First, mRNAs encoding fungal antigens (blue) or genetic adjuvants (red) are synthesized as follows: *in vitro* synthesis of a codon optimized fungal antigen gene, PCR amplification of this linear DNA template with the addition of a T7 promoter and Poly(A) tail, and subsequent *in vitro* transcription of the base modified polyadenylated mRNAs. Then the mRNAs are encapsulated in lipid nanoparticles (LNPs). The mRNA-LNPs are administered to the host and internalized via endocytosis by antigen presenting cells (APCs), including macrophages or dendritic cells. The released mRNAs are translated into proteins by host ribosomes. The translated genetic adjuvants are secreted to modulate the immune response. Translated antigens can activate the immune system primarily in two ways: (1) Cytosolic proteins are degraded by proteasomes to generate antigenic epitopes, which are presented by major histocompatibility complex class I (MHC-I) molecules of APCs. The MHC-I with the epitope binds to the cognate T cell receptor to activate antigen-specific CD8^+^ T cells for their cytotoxic effect through the secretion of perforin and granzyme. (2) Secreted or membrane proteins are internalized through endocytosis by APCs and degraded into peptide epitopes, which are subsequently presented on the cell surface via MHC-II molecules for recognition by CD4^+^ T cells. This can activate both the cellular immune response by secreting cytokines and the humoral immune responses by co-activating B cells. Created in BioRender.

Over the past decade, some major technological innovations and research investments have enabled mRNAs to become a promising therapeutic tool in vaccine development [10–12]. Here, we briefly discuss several advantages of the mRNA platform: safety, efficacy, scalability, and flexibility. **First**, there are safety concerns for DNA-based vaccines due to potential integration into host genomes, for whole-cell vaccines due to risks of cross-reactivity to host molecules or pathogenicity of live attenuated vaccines, and for recombinant protein vaccines due to undesired effects of potential contaminants from bacteria or cell cultures in the purification process [13]. By contrast, mRNA is a non-integrating, degradable, and non-infectious platform [14]. Additionally, *in vitro* transcription of mRNA is a cell-free process, which avoids the cell-derived impurities [14]. **Second**, efficacy of recombinant protein vaccines could be restricted by lacking parts of the native proteins or proper posttranslational modifications. These are important factors to consider given that most fungal antigens are extracellular proteins which are often glycosylated and membrane-bound. In-host translation of mRNA vaccines allows eukaryotic post-translational processing and modifications, which resemble the native pathogen-derived antigens. For example, Pvs25 is a glycosylphosphatidylinositol-anchored membrane protein of the malaria parasite *Plasmodium vivax*. The Pvs25 mRNA vaccines yielded higher antibody response than the Pvs25-based truncated recombinant protein vaccine [15, 16]. Furthermore, the full-length version of Pvs25 mRNA vaccine showed higher efficacy against transmission of *P. vivax* than the truncated versions lacking the C-terminal membrane anchor*,* suggesting the membrane protein expressed in the host could enhance the immune response [15]. **Third**, ideal vaccine manufacturing should be flexible and scalable. The high yields of *in vitro* transcription to generate mRNAs allow scalable manufacturing of mRNA vaccines [14]. Moreover, it is easier to change or add antigens using the mRNA platform [17], which is critical for developing multivalent vaccines against complex cellular pathogens like fungi or parasites (Fig 1).

However, there are some barriers for fungal mRNA vaccine development. **First**, the cost of mRNA vaccine is high compared to others. For example, the US government supported the development of the SARS-CoV-2 mRNA vaccine and obtained a lower price (BioNTech $19.5/dose and Moderna $15.0/dose) during the pandemic, but the private price is $141.70/dose [18]. In comparison, the price of non-replicating viral vector vaccine from Johnson & Johnson for the US and protein-based SARS-CoV-2 vaccine from Novavax for SARS-CoV-2 Vaccines Global Access (COVAX) was $10.0/dose and $3/dose, respectively [19]. The cold chain requirement for mRNA vaccines would also be an issue for resource-limited countries. Both Moderna and BioNTech mRNA vaccines are stable up to 6 months at −25 °C, 30 days at 4 °C, and 6 h at room temperature. **Second**, the duration of protection by mRNA vaccines is short. Most studies found that protection waned significantly within 3–6 months after vaccination, especially against the Omicron variant [20]. **Third**, posttranslational modifications of antigens occurring in host systems may not fully recapitulate their native counterparts. One noticeable divergence is glycosylation, where glycosylation sites remain conserved between fungi and mammals, but the resultant glycan structures exhibit significant structural divergence [21]. **Finally**, vaccine hesitancy, particularly toward mRNA vaccines, could undermine efforts to combat fungal infections [22].

## 2. What is the critical factor to maximize in-host expression of mRNAs?

The effective patient dose of mRNA vaccines ranges widely from 2 to 100 μg [12,23]. Vaccination of mice with 0.1–1 μg mRNA encoding the spike protein is fully protective against SARS-CoV-2 in mouse models [24]. To reduce the cost and potential adverse effects, it is important to use a low but effective dose, which necessitates the optimization of the in-host translation of mRNA vaccines.

Synthetic mRNAs have the same structure as natural mRNAs including a 5′ cap, 5′ and 3′ untranslated regions (UTRs), and a 3′ poly(A) tail flanking the coding sequence of interest (Fig 1). The 5′ and 3′ UTRs regulate mRNA translation and maintain mRNA stability, and thus they are critical for the success of mRNA vaccines. The UTRs from highly expressed genes, such as the hemoglobin subunit α or β genes, are preferred for synthetic mRNAs [25]. However, the impact of UTRs on translation of mRNA vaccines can vary by cell type [26–30]. Using the 5′ and 3′ UTRs from human hemoglobin subunit beta (*HBB*) as reference, Leppek and colleagues assessed the translation efficiencies of 112 UTRs in human embryonic kidney-derived HEK293T cells [28]. They found that the 5′ UTRs from mouse *COL1A2*, *Hoxa9* and *Rpl18a*, plant *RBCS1A*, and plant virus *TEV* and *TMV* had a higher ribosome load than *HBB* [28]. Cao and coworkers used an algorithm to obtain synthetic 5′ UTRs, screened 12,000, and identified three that enhanced protein expression across a variety of cell types, including HEK293T cells, human prostate cancer-derived PC3 cells, and human muscle tissue [27]. Fusion of two synthetic 5′ UTRs further enhanced the protein expression [27]. Niessen and colleagues found that the recombinant 3′ UTRs generated from the amino-terminal enhancer of split gene and that of mitochondrially encoded 12S rRNA gene enhanced translation and immune responses in mice [29]. Recently, a review article summarized current UTR sequences of SARS-CoV-2 mRNA vaccines from different manufacturers [23]. We tested five different 5′ UTR sequences of SARS-CoV-2 mRNA vaccines in murine macrophage cells and found that the modified 5′ UTR from the hemoglobin subunit alpha (*HBA1*) used in the BioNTech mRNA vaccine and the 5′ UTRs from *HBB* significantly enhanced the expression compared to the 5′ UTR from *ACTB* (cytoplasmic actin) [31]. These studies illustrate the importance of optimizing 5′ and 3′ UTRs to maximize the translation of mRNA vaccines.

## 3. What factors affect the delivery of mRNA vaccines?

The fundamental mechanism of mRNA vaccine technology is based on a lipid nanoparticle (LNP) vehicle that delivers mRNAs encoding the antigen into the target host cell, allowing the host cell to express the antigen to elicit the immune response (Fig 1). Thus, the site of delivery and the trafficking of the LNPs impact the efficacy of the vaccines. By monitoring the dynamics of mRNA vaccine labeled with radionuclide-near-infrared probes after intramuscular injection to cynomolgus macaques via positron emission tomography–computed tomography (PET–CT) and near-infrared imaging, Santangelo and colleagues found that mRNA vaccines traveled to the lymph nodes near the injection sites, but they rarely reached distal lymph nodes [32]. The size of the LNPs affects the trafficking of mRNA vaccine. Nanoparticles of 10–100 nm are suitable for traveling through lymphatic vessels to lymphoid tissues, while those of 100–200 nm exhibit poor migration to lymph nodes [33]. Nanoparticles larger than 200 nm stay at the injection site and rely entirely on uptake by APCs for trafficking [33]. However, the size of LNPs is restrained by its capacity of mRNA packaging. 100 nm LNPs generally only contain 2–3 mRNAs of 1,900 nt [34]. Thus, it is critical to consider the size of LNPs for trafficking and mRNA packaging.

The size of LNPs may be partly controlled by lipid composition [34, 35]. LNPs are typically composed of four components: ionizable lipids, helper phospholipids, cholesterol, and PEGylated lipids [11]. The average LNP diameter without mRNAs decreases from 210 to 100 nm when the lipid-conjugated polyethylene glycol (DMG-PEG2000) increases from 0.25% to 3% if the other lipid components remain the same [34]. Besides the size of LNPs, the surface properties also influence LNPs trafficking in the host. Coating the LNP surface with hydrophilic stealth materials, such as DMG-PEG2000, enabled the translocation of over 100 nm-sized LNPs from the injection site to lymph nodes and increased the uptake by APCs [36, 37]. Thus, optimization of the lipid components and surface properties of the nanoparticles will be helpful for improving mRNA vaccine efficacy.

The route of administration also can influence outcomes of mRNA vaccines [14]. Most mRNA vaccines are delivered via the intramuscular or the subcutaneous route, which provide slow and sustained release [38]. Due to a lower risk of adverse effects, the ability to induce a robust immune response, and the simplicity of delivery, intramuscular administration is the predominant route for mRNA vaccination in the clinical setting [39, 40]. Intradermal administration has been considered because skin contains more immune-related cells compared to muscle and subcutaneous tissues [41]. Indeed, mRNA vaccines administered by intradermal injection resulted in more robust humoral responses [42, 43]. Intradermal administration of 10 or 20 μg of mRNA-1273 SARS-CoV-2 is as effective as intramuscular injection of 100 μg of the same mRNA vaccines in terms of production of spike protein-specific antibodies [44]. Likewise, intradermal injection of one-fifth BNT162b2 SARS-CoV-2 vaccine (6 μg mRNA) as a booster dose induced comparable humoral and cellular immune responses as intramuscular injection of the full dose (30 μg mRNA) [45, 46]. Despite these promising findings, the intradermal route is less frequently used due to technical difficulties in achieving an accurate injection in clinical practice [40]. Nonetheless, intradermal administration should be considered for fungal mRNA vaccine delivery, and improvement in delivery technologies such as microneedle array patches could potentially make intradermal injection practical and accurate [47].

## 4. Can adjuvants improve fungal mRNA vaccines?

Adjuvants enhance the immunogenicity of vaccines when administered in conjunction with antigens [48, 49]. Adjuvants have become increasingly important in recombinant protein or nucleic acid vaccines, which lack many of the pathogen-associated molecular patterns presented in conventional live-attenuated and inactivated whole-cell vaccines [48, 49]. mRNA vaccines are considered inherently self-adjuvanted due to the exogenous nucleoside-unmodified mRNA and the lipid components of LNPs [48, 49]. However, that inherent immunostimulatory capacity of mRNA-LNPs may not induce the desired immunity or at a sufficient amplitude. Consequentially, the inherent immunity elicited by LNPs is polarized toward inflammatory innate immune responses, resulting in low T helper cell-driven memory responses and short-lived immunity. This necessitates frequent booster immunizations or the addition of a specific adjuvant. Compared to mRNA vaccines against acellular viruses, which themselves are often protective, mRNA vaccines against eukaryotic organisms may require adjuvants. For example, we found the mRNA vaccine encoding the cryptococcal antigen Cda1 (chitin deacetylase 1) alone does not provide strong protection against cryptococcosis [31]. However, when *CDA1*-LNPs were combined with purified capsule, which is predominantly composed of polysaccharides (>97%), the vaccine provides significantly enhanced protection against cryptococcal infection [31]. Similarly, the *RPL6*-LNP mRNA vaccine alone was ineffective in mice against the sporozoites of the protist pathogen *Plasmodium berghei*. However, the addition of the adjuvant α-galactosylceramide resulted in effective protection [50]. Therefore, it is crucial to consider and select the appropriate adjuvant in fungal mRNA vaccines to achieve the desired immune response.

There are two main types of adjuvants: classical adjuvants and genetic adjuvants [48]. Classical adjuvants, which are often chemical compounds, have been explored to enhance the immunogenic potential of mRNA vaccines [51]. For example, the arginine-rich protamine peptides can activate TLR7/8 pathways to elicit B- and T-cell-dependent response in the mRNA vaccines against influenza A or tumors [52]. The cholesterol-modified cationic peptide DP7 improves the immune responses and is used in a cancer mRNA vaccine [53]. C16-R848, the palmitic acid-modified TLR7/8 agonist Resiquimod (R848), induces an effective anti-tumor immunity in mice [54].

Genetic adjuvants are commonly used in DNA vaccines due to the chronological incompatibility between the immediate effect of classical adjuvants and the delayed expression of DNA-encoded antigens [49]. Genetic adjuvants are just beginning to be explored for mRNA vaccines, mostly for cancer. For example, mRNAs encoding a constitutively active allele of STING (STING^V155M^) amplify antigen-specific CD8^+^ T cell response in an mRNA cancer vaccine [55]. An mRNA encoding a fused protein of a tumor antigen and an antibody targeting CD3 induces sustained endogenous synthesis of the bispecific T cell engaging antibodies for eliminating advanced tumors [56]. Recently, genetic adjuvants are also being explored for vaccines against infectious diseases. An mRNA encoding the human Fc-conjugated receptor binding domain is used as an adjuvant in a SARS-CoV-2 mRNA vaccine, which increases the production of neutralizing antibodies in the transgenic mouse model [57]. An mRNA encoding IL-12p70 amplifies cellular and humoral immune responses of the BNT162b2 SARS-CoV-2 mRNA vaccine, which allows reduced dosing to achieve the same antibody response [58]. Given that the genetic adjuvants enable the spatiotemporally simultaneous expression of antigens and immunomodulators of the mRNA vaccine (Fig 1), they could be extremely valuable for the success of mRNA vaccines against fungal diseases.

It is important to note that the ideal adjuvant should enhance vaccine immunogenicity without compromising tolerability or safety. Unfortunately, adjuvant development has not kept pace with advancement in other vaccine areas, resulting in a very limited number of adjuvants approved for human use due to reactogenicity or potential adverse effects [59]. Thus, comprehensive investigations in both the mode of action and potential toxicity of new adjuvants should be a priority for future vaccine research.

## 5. How can mRNA vaccines be optimized for immunocompromised hosts?

With the AIDS epidemic and advanced medical treatments (e.g., transplant and cancer therapy), the number of immunocompromised individuals has risen, increasing the global burden of fungal diseases [60]. Although current fungal vaccines often provide protection in immunocompetent hosts, they are not as effective in immunocompromised hosts during preclinical studies in mice [1,13]. Similarly, the efficacy of COVID-19 mRNA vaccines is generally lower in immunocompromised individuals. In a clinical trial of the BNT162b2 mRNA vaccine in immunocompromised patients (180 HIV^+^, 90 CAR T cell therapy, 89 solid organ transplantation, and 90 chronic lymphocytic leukemia), 72.2% seroconverted compared to 100% in healthy people [61]. This result is consistent with a systematic review and meta-analysis of SARS-CoV-2 mRNA vaccine efficacy in immunocompromised patients in terms of seroconversion rates [62]. In another study, vaccination with the SARS-CoV-2 mRNA vaccine in kidney transplant recipients displayed reduced specific CD4^+^/CD8^+^ T cell frequencies and neutralizing antibody response [63]. Based on hospitalization data, the effectiveness of mRNA vaccination was lower among immunocompromised adults (77%) than among immunocompetent adults (90%) [64]. A second dose was associated with consistently improved seroconversion across all patient groups, albeit at a lower magnitude for organ transplant recipients. Taken together, mRNA vaccines for immunocompromised people may require increased booster shots.

As discussed previously, the SARS-CoV2 mRNA vaccine induces relatively weak CD4^+^ T cell response [65]. Given the advantage of co-delivery of mRNAs encoding CD4^+^ T cell stimulating-adjuvants, this platform might be particularly effective in augmenting the efficacy of mRNA vaccines in immunocompromised patients. Dowling and colleagues creatively designed single-chain IL-12p70 as adjuvant with the SARS-CoV2 mRNA vaccine [58]. Vaccination of this combination amplified the spike protein-specific immunity and increased Th1 polarization in mice [58]. Furthermore, IL-12p70 adjuvanted BNT162b2 immunization strongly induced CD4^+^ and CD8^+^ T cell response in aged mice [58]. The promising pre-clinical data suggest that co-delivery of mRNAs encoding CD4^+^ T cell stimulating-adjuvant with mRNA vaccine may work for the immunocompromised patient.

IFN-γ/IL-12/TNFα cytokines produced by type 1 T helper (Th1) cells are associated with protection against cryptococcosis and IFN-γ is essential for host defense. In a phase 2, double-blind, placebo-controlled trial, administration of rIFN-γ1b three times weekly (100 or 200 μg dose) to the standard care helped more HIV patients convert from cryptococcal positive to negative [66]. Hence, a forthcoming goal for fungal mRNA vaccine development will be using mRNAs encoding IFN-γ and other cytokines as genetic adjuvants to enhance the efficiency of mRNA vaccines in immunocompromised individuals. Additionally, tailoring the combination of different cytokines to maximize protection against different fungal diseases in different patient populations may be a valuable pursuit.

## 6. Will a heterologous vaccine regimen combining mRNA vaccine and subunit protein vaccine boost immune responses against fungal infections?

A homologous prime-booster immunization strategy means the same vaccine is given in the prime and subsequent booster immunizations [67]. As different vaccines for the same disease become available, the heterologous prime-booster immunization strategy has increasingly become a possibility. Over the past decade, studies have shown that heterologous prime-booster immunizations or unmatched vaccine delivery methods using the same antigen could be more effective than the homologous prime-booster format, including those against HIV, influenza, polio, and hepatitis viruses [67–70]. The observation that a heterologous prime-booster vaccination strategy against SARS-CoV-2 variants is more effective than a homologous strategy prompted the WHO to issue an interim recommendation for heterologous SARS-CoV-2 vaccine schedules combining different vaccine platforms during the COVID-19 pandemic [71–73]. Zhang and colleagues found that protein vaccine prime-mRNA vaccine booster induces modestly higher serum neutralizing activity, while mRNA vaccine prime-protein vaccine booster induced more robust Th1 cellular response than the homologous immunization of mRNA or protein vaccines [74]. The heterologous protein/mRNA immunization strategy further enhanced antibody responses against various COVID variants [74]. In another study, priming intramuscularly with a SARS-CoV-2 mRNA vaccine followed by boosting intranasally with a recombinant protein vaccine induced stronger mucosal and systemic antibody responses compared to homologous regimens [75]. In addition to viral vaccines, combinations of mRNA and subunit protein vaccines have also been hypothesized to boost immune responses against parasite diseases [76, 77]. For example, homogenous vaccination with the LEISH-F2 mRNA vaccine or homogenous vaccination with recombinant LEISH-F2 protein did not provide protection against *Leishmania donovani*, but a heterogenous vaccination with the mRNA vaccine as the prime and recombinant protein vaccine as the booster significantly reduced parasite burden [77]. Therefore, it will be useful to test the heterologous immunization strategy for fungal vaccines.
